# Association of Glucose-Lowering Therapy with Myocardial Work Recovery and Reverse Remodeling After STEMI

**DOI:** 10.3390/jcm15134891

**Published:** 2026-06-23

**Authors:** Bogdan-Flaviu Buz, Venkata Sai Harshabhargav Chenna, Ankit Sharma, Pravallika Myneni, Iulia Georgiana Bogdan, Cristian Mornos, Simina Crisan, Dan Gaita, Constantin-Tudor Luca, Diana-Aurora Arnautu, Camelia Gurban, Felicia Marc, Florina Caruntu, Minodora Andor

**Affiliations:** 1 Faculty of Medicine, Doctoral School, Victor Babes University of Medicine and Pharmacy, 300041 Timisoara, Romania; flaviu-bogdan.buz@umft.ro (B.-F.B.); andor.minodora@umft.ro (M.A.); 2 Multidisciplinary Heart Research Center, Victor Babes University of Medicine and Pharmacy, 300041 Timisoara, Romania; aurora.bordejevic@umft.ro (D.-A.A.);; 3 Department of Internal Medicine, Victor Babes University of Medicine and Pharmacy, 300041 Timisoara, Romania; 4 Department of Internal Medicine, Trinity Health Oakland/Wayne State University School of Medicine, 44405 Woodward Avenue, Pontiac, MI 48341, USA; 5 Faculty of General Medicine, Yerevan State Medical University After Mkhitar Heratsi, 2 Koryun Street, Yerevan 0025, Armenia; ankitsharmanarangpur@gmail.com; 6 Katuri Medical College and Hospital, Katuri Nagar, Chinakondrupadu, Guntur 522019, Andhra Pradesh, India; myneni2737@gmail.com; 7 Discipline of Infectious Diseases, Faculty of Medicine, Victor Babes University of Medicine and Pharmacy Timisoara, Eftimie Murgu Square 2, 300041 Timisoara, Romania; iulia-georgiana.bogdan@umft.ro; 8 Timisoara Municipal Clinical Emergency Hospital, 300040 Timisoara, Romania; mornos.cristian@umft.ro; 9 Department of Cardiology, Victor Babes University of Medicine and Pharmacy, 300041 Timisoara, Romania; simina.crisan@umft.ro (S.C.); dan.gaita@umft.ro (D.G.); constantin.luca@umft.ro (C.-T.L.); 10 Timisoara Institute of Cardiovascular Diseases, 13 Gheorghe Adam Street, 300310 Timisoara, Romania; 11 Department of Biochemistry and Pharmacology, Victor Babes University of Medicine and Pharmacy Timisoara, Eftimie Murgu Square 2, 300041 Timisoara, Romania; 12 Department of Medical Sciences, Faculty of Medicine and Pharmacy, University of Oradea, 410073 Oradea, Romania

**Keywords:** myocardial infarction, ventricular remodeling, sodium–glucose transporter 2 inhibitors, glucagon-like peptide-1 receptor agonists, echocardiography

## Abstract

**Background:** Patients with type 2 diabetes mellitus (T2DM) who present with ST-segment elevation myocardial infarction (STEMI) remain at high risk of adverse remodeling after reperfusion. This observational study examined whether pre-admission glucose-lowering therapy class was associated with six-month left ventricular (LV) reverse remodeling and myocardial work recovery. **Methods:** We analyzed 253 patients with STEMI, baseline LV ejection fraction ≤ 50%, successful primary PCI, and complete baseline and six-month echocardiography. The primary inferential analyses focused on 75 patients with T2DM, grouped according to pre-admission therapy with SGLT-2 inhibitors, GLP-1 receptor agonists, DPP-4 inhibitors, or conventional therapy; non-diabetic patients were retained as a descriptive reference group. Clinical outcome, propensity-score, subgroup, and mediation analyses were considered exploratory because of small subgroup and event counts. **Results:** SGLT-2 inhibitor and GLP-1 receptor agonist exposure was associated with larger improvements in LVEF, LV volumes, and global work efficiency than DPP-4 inhibitors or conventional therapy. Crude MACE rates were highest in the conventional-therapy group, but event estimates were imprecise and confounded by baseline risk, revascularization status, and discharge therapy. **Conclusions:** In patients with T2DM recovering from STEMI, pre-admission exposure to SGLT-2 inhibitors and, to a lesser extent, GLP-1 receptor agonists was associated with more favorable structural and myocardial work recovery. These hypothesis-generating findings should be interpreted as associations and require confirmation in adequately powered prospective studies.

## 1. Introduction

ST-segment elevation myocardial infarction (STEMI) remains a leading cause of heart failure hospitalization and all-cause mortality worldwide, despite the widespread availability of reperfusion therapy by primary percutaneous coronary intervention (PCI) [1,2]. The subset of patients presenting with reduced left ventricular ejection fraction (LVEF) at admission is particularly prone to adverse left ventricular (LV) remodeling, defined by progressive cavity dilatation, wall thinning, and diminished contractile performance [3]. Approximately half of this group develops a more favorable structural response, reverse remodeling, which translates into markedly lower rates of major adverse cardiac events (MACEs) and late heart failure [4,5].

Type 2 diabetes mellitus (T2DM) complicates STEMI in roughly one-third of cases and worsens every post-infarct outcome, including mortality, heart failure incidence, and reinfarction [6]. The mechanisms are multiple and include the following: microvascular rarefaction, impaired mitochondrial substrate flexibility, interstitial fibrosis, autonomic dysregulation, and a pro-inflammatory milieu that extends infarct size and attenuates repair [7,8]. A recent systematic review by our group synthesized evidence that hypoglycemic medications differ widely in their effect on LV reverse remodeling, with sodium–glucose co-transporter-2 inhibitors (SGLT-2is) and glucagon-like peptide-1 receptor agonists (GLP-1 RAs) showing the most consistent benefits across heart failure and diabetic cardiomyopathy populations [9].

SGLT-2 inhibitors reduce hospitalization for heart failure in patients with and without diabetes, and randomized trials have shown reductions in LV end-diastolic volume, modest gains in ejection fraction, and improvements in diastolic filling within three to twelve months [10,11]. Their cardioprotective signal extends beyond glycemic control and is attributed to natriuresis, reduced preload, enhanced ketone substrate utilization, modulation of sodium–hydrogen exchange, and suppression of cardiac fibrosis [12]. Recent AMI-specific evidence, however, remains cautious. An updated 2025 meta-analysis of early SGLT-2 inhibitor initiation after acute MI found lower hospitalization for heart failure and MACEs in frequentist analyses, but Bayesian estimates retained uncertainty and mortality endpoints were not significantly reduced [13]. A recent double-blind STEMI trial of dapagliflozin assessed myocardial fibrosis after primary PCI and provides important randomized STEMI-specific context, underscoring that structural recovery signals require prospective confirmation [14]. GLP-1 receptor agonists share complementary biology through endothelial nitric oxide release, reduced oxidative stress, and anti-inflammatory effects; by contrast, DPP-4 inhibitors have shown a more modest and inconsistent signal, and neutral trials dominate the evidence base for conventional therapy built on metformin, sulfonylureas, and insulin [13,14].

Beyond LVEF and cavity dimensions, advanced echocardiographic tools have emerged that characterize post-infarct recovery in unprecedented detail. Two-dimensional speckle-tracking echocardiography yields global longitudinal strain, a load-dependent measure of myocardial deformation that detects subclinical dysfunction at the preserved LVEF [15]. More recently, non-invasive pressure–strain analysis produced the myocardial work indices, global work index (GWI), global constructive work (GCW), global wasted work (GWW), and global work efficiency (GWE), which integrate afterload into the assessment of mechanical performance and provide a load-independent window into cardiac energetics [16]. In our recent cohort study of 253 reperfused STEMI patients with baseline LVEF ≤ 50%, baseline GWE emerged as the strongest independent predictor of reverse remodeling, outperforming traditional demographic and hemodynamic variables [17].

The intersection of hypoglycemic therapy class and myocardial work dynamics after STEMI has not, to our knowledge, been examined. Such an analysis is conceptually important because myocardial work indices may provide an early, afterload-integrated marker of post-infarct mechanical recovery. Nevertheless, treatment allocation in routine care is strongly influenced by diabetes duration, renal function, hemodynamic status, body habitus, frailty, and physician preference; therefore, any class differences in an observational cohort must be interpreted as associations rather than evidence of therapeutic causality.

We aimed to examine whether pre-admission glucose-lowering therapy class was associated with six-month LV reverse remodeling and myocardial work recovery after reperfused STEMI in patients with T2DM. Clinical events, propensity-score analyses, subgroup analyses, and mediation analyses were pre-specified as secondary and exploratory because of the limited diabetic subgroup size and small number of events.

## 2. Materials and Methods

### 2.1. Study Design and Population

This was a single-center cohort analysis of prospectively collected data from consecutive STEMI patients treated at the Timisoara Institute of Cardiovascular Diseases between 20 March 2023 and 31 August 2024. Inclusion criteria mirrored those of our parent cohort and comprised: (i) age ≥ 18 years; (ii) STEMI confirmed per the Fourth Universal Definition of Myocardial Infarction; (iii) successful primary PCI within 24 h of symptom onset, defined as Thrombolysis in Myocardial Infarction (TIMI) grade 3 flow in the infarct-related artery; (iv) an echocardiographically determined LVEF ≤ 50% within 48 h of PCI; and (v) availability of complete baseline and six-month echocardiographic studies with image quality adequate for speckle-tracking analysis. Exclusion criteria were previous myocardial infarction, known cardiomyopathy, significant native or prosthetic valvular disease, atrial fibrillation at the baseline examination, hemodynamic instability precluding transthoracic imaging, and suboptimal echocardiographic windows. All participants provided written informed consent, and the institutional ethics committee of Victor Babes University of Medicine and Pharmacy Timisoara approved the protocol (approval no. 10, 10 March 2023) in accordance with the Declaration of Helsinki.

Before final analytic cohort definition, 256 reperfused STEMI patients with baseline LVEF ≤ 50% and complete baseline and six-month echocardiography were screened. Seventy-eight had physician-confirmed T2DM documented in their pre-admission records, with a minimum disease duration of six months and a minimum exposure of three months to index hypoglycemic therapy. Three patients used two index drug classes simultaneously before admission and were excluded before therapy-class assignment because a single exposure category could not be assigned unambiguously. The final analytic cohort therefore included 253 patients: 178 without T2DM and 75 with T2DM. The T2DM subgroup was divided into four mutually exclusive pre-admission therapy classes: (i) SGLT-2 inhibitor monotherapy or combination including dapagliflozin or empagliflozin (*n* = 22); (ii) GLP-1 receptor agonist monotherapy or combination including liraglutide or semaglutide (*n* = 14); (iii) DPP-4 inhibitor monotherapy or combination including sitagliptin or linagliptin without SGLT-2 inhibitor or GLP-1 receptor agonist exposure (*n* = 16); and (iv) conventional therapy with metformin, sulfonylureas, and/or insulin alone (*n* = 23). The 178 patients without T2DM, pre-diabetes, or hypoglycemic therapy served only as a descriptive reference group and were not treated as a therapeutic comparator for causal inference.

Screened STEMI patients with successful primary PCI, baseline LVEF ≤ 50%, and complete echocardiographic follow-up (*n* = 256) were excluded before therapy-class assignment because of simultaneous exposure to two index glucose-lowering classes (*n* = 3). The final analytic cohort (*n* = 253) included: a non-diabetic descriptive reference group (*n* = 178) and T2DM analytic subgroup (*n* = 75), comprising SGLT-2 inhibitor (*n* = 22), GLP-1 receptor agonist (*n* = 14), DPP-4 inhibitor (*n* = 16), and conventional therapy (*n* = 23).

### 2.2. Echocardiographic Assessment and Myocardial Work Analysis

Transthoracic echocardiography was performed within 48 h of the index PCI and again at the six-month clinical review using a Vivid S5 ultrasound system (GE Healthcare, Chicago, IL, USA) with a 1.5–4.5 MHz phased-array transducer. Parasternal long-axis, short-axis, and apical four-, two-, and three-chamber views were acquired at a frame rate of 50–80 Hz. LV end-diastolic and end-systolic volumes, as well as LVEF, were calculated using the biplane modified Simpson method per American Society of Echocardiography guidelines; the stroke volume index was derived by dividing stroke volume by body surface area. Wall motion score index was scored by two experienced readers blinded to hypoglycemic therapy class. Reverse remodeling was defined a priori as a reduction of ≥ 15% in LV end-diastolic volume (LVEDV) between baseline and six-month examinations; an indexed LVEDV threshold of ≥10% reduction was used as a sensitivity definition and showed 91% concordance (kappa = 0.89) with the primary definition.

Two-dimensional speckle-tracking echocardiography was performed offline on EchoPAC (version 203, GE Healthcare) after manual adjustment of the endocardial border. Global longitudinal strain was obtained by averaging peak systolic strain values across all seventeen LV segments from the apical views. Myocardial work indices were computed from non-invasive pressure–strain loops using brachial cuff blood pressure measured at the moment of image acquisition as a surrogate for peak LV pressure. The four derived parameters were global work index (GWI, total myocardial work during systole), global constructive work (GCW, work contributing to ejection), global wasted work (GWW, dissipated energy from segmental lengthening during systole and shortening during isovolumetric relaxation), and global work efficiency (GWE, GCW/(GCW + GWW) × 100). To verify reproducibility, twenty-five randomly selected studies were reanalyzed by the same certified operator; intra-observer coefficients of variation were 4.1% for GLS, 5.3% for GWI, 4.7% for GCW, and 3.9% for GWE, with intraclass correlation coefficients between 0.91 and 0.96.

### 2.3. Hypoglycemic Therapy Classification, Laboratory Data, and Clinical Follow-Up

Pre-admission hypoglycemic therapy was abstracted from electronic discharge letters from the referring primary-care physician or endocrinologist, cross-checked against the National Electronic Prescription Registry, and confirmed with the patient or caregiver at the baseline visit. Treatment duration, dose, and adherence (self-reported Morisky-4 score) were recorded. Post-discharge diabetes therapy was also abstracted at discharge and at the six-month follow-up, including continuation, discontinuation, initiation, and switching of SGLT-2 inhibitors, GLP-1 receptor agonists, DPP-4 inhibitors, insulin, metformin, or sulfonylureas. The primary analysis retained patients in their pre-admission class to avoid immortal-time and post-index treatment bias; a per-protocol sensitivity analysis was limited to patients who remained on the same therapy class through six months. PCI strategy was reviewed for all patients with multivessel coronary artery disease. Complete revascularization was defined as successful treatment of all angiographically significant non-culprit lesions during index PCI or staged PCI before the six-month echocardiographic assessment; residual unrevascularized disease was recorded when additional revascularization was clinically indicated but not completed before follow-up.

Clinical follow-up was conducted at one, three, six, and twelve months post-PCI and every six months thereafter, with a median total follow-up of 17 months (interquartile range of 14–22). The primary structural endpoint was reverse remodeling, as defined above. The primary clinical endpoint was a composite of MACEs, comprising all-cause mortality, hospitalization for heart failure, and non-fatal reinfarction; each component was adjudicated by two independent cardiologists blinded to therapy class, with disagreements resolved by a third adjudicator. Secondary endpoints were the individual MACE components, change from baseline in each echocardiographic parameter, and a composite safety endpoint (genitourinary infection, diabetic ketoacidosis, severe hypoglycemia, acute kidney injury). Guideline-directed medical therapy at discharge, renin–angiotensin system blockade, beta-blockers, mineralocorticoid receptor antagonists, and high-intensity statins were prescribed at the treating cardiologist’s discretion and documented in the electronic record.

### 2.4. Statistical Analysis

Continuous variables were tested for normality with the Shapiro–Wilk test and presented as mean ± standard deviation or median with interquartile range, as appropriate. Categorical variables are expressed as counts with percentages. Between-group comparisons across the five descriptive groups used one-way ANOVA with Tukey HSD post hoc testing for normally distributed continuous variables, Kruskal–Wallis tests with Dunn–Holm correction for non-parametric variables, and chi-square or Fisher exact tests with false-discovery-rate correction for pairwise categorical contrasts. Pairwise post hoc results are reported for variables with significant omnibus *p*-values in the table footnotes or supplementary post hoc table.

The primary inferential analyses were restricted to the T2DM subgroup and emphasized univariable associations because only 75 patients were distributed across four therapy classes. Multivariable logistic regression for reverse remodeling was retained only as a parsimonious exploratory sensitivity analysis using a limited number of pre-specified covariates selected for clinical relevance (baseline GWE and Killip class), without automated backward elimination. Clinical outcome analyses used Kaplan–Meier curves, log-rank tests, and univariable Cox models as the main approach; parsimonious Cox sensitivity models were limited to baseline GWE and Killip class because of the small number of events. Propensity-score matching, mediation, and subgroup analyses were considered exploratory and hypothesis-generating. Mediation terminology was changed from causal mediation to exploratory mediation because treatment allocation was not randomized and changes in GWE and LVEDV occurred over the same follow-up interval. A two-sided *p*-value < 0.05 was considered statistically significant. All analyses were performed with R 4.3.2 and MedCalc 23.2.6.

## 3. Results

Among the 118 patients with multivessel coronary artery disease, complete index or staged revascularization before the six-month echocardiographic assessment was documented in 48/71 non-diabetic patients (67.6%), 9/12 SGLT-2 inhibitor patients (75.0%), 6/9 GLP-1 receptor agonist patients (66.7%), 7/11 DPP-4 inhibitor patients (63.6%), and 8/15 conventional-therapy patients (53.3%). Thus, residual unrevascularized multivessel disease was numerically most frequent in the conventional-therapy group, which may have contributed to less favorable remodeling and clinical outcomes.

Post-discharge diabetes therapy was available for all 75 patients with T2DM. By six months, 20/22 SGLT-2 inhibitor users, 13/14 GLP-1 receptor agonist users, 14/16 DPP-4 inhibitor users, and 17/23 conventional-therapy patients remained on their pre-admission class. During follow-up, two SGLT-2 inhibitor users discontinued therapy because of renal function or tolerability concerns, one GLP-1 receptor agonist user discontinued therapy, two DPP-4 inhibitor users switched to an SGLT-2 inhibitor, and four conventional-therapy patients initiated a contemporary class (three SGLT-2 inhibitors and one GLP-1 receptor agonist). These changes support the intention-to-treat exposure definition and the need for cautious interpretation of six-month remodeling by pre-admission therapy class.

Table 1 summarizes the baseline characteristics of the 253 STEMI patients stratified by pre-admission hypoglycemic therapy. The four diabetic subgroups showed clinically important heterogeneity consistent with real-world prescribing patterns and confounding by indication. Patients on DPP-4 inhibitors were the oldest and had the lowest estimated glomerular filtration rate, whereas patients on GLP-1 receptor agonists had the highest body mass index. Glycemic control worsened progressively from SGLT-2 inhibitor therapy to conventional therapy, and diabetes duration followed the same gradient. The conventional-therapy group also had the highest proportion of Killip class ≥ III and the highest NT-proBNP, indicating a substantially higher-risk presentation. Multivessel coronary artery disease was more frequent in diabetic patients than in the non-diabetic reference group, and residual unrevascularized disease was numerically most common in conventional-therapy patients. These imbalances were considered central to the interpretation of all subsequent outcome analyses and motivated the use of cautious, association-based language throughout the manuscript.

At baseline, conventional echocardiographic indices were similar across therapy groups (Table 2). LVEF ranged narrowly from 40.3 ± 7.2% in the SGLT-2i group to 41.8 ± 6.7% in the non-diabetic reference (*p* = 0.871), and LV end-diastolic volumes differed by only 7.5 mL between the extremes (*p* = 0.563). Wall motion score index (*p* = 0.374) and global longitudinal strain (*p* = 0.247) did not discriminate between the groups, confirming that the classical measures of chamber geometry and deformation captured a common level of acute ischemic dysfunction across the entire cohort. Global constructive work was lowest in the conventional-therapy group (1714 ± 206 mmHg%) and highest in the non-diabetic reference (1867 ± 172 mmHg%, *p* = 0.017). Global wasted work followed the opposite gradient (247.8 ± 52.6 in conventional vs. 208.3 ± 41.6 mmHg% in non-diabetic, *p* = 0.003), yielding a lower global work efficiency in the DPP-4i (80.6%) and conventional (80.1%) strata compared with non-diabetic (83.1%, *p* = 0.048 across groups). E/e′ ratios also differed significantly (*p* = 0.002), with conventional-therapy patients showing the highest filling-pressure surrogate. These baseline energetic and diastolic differences provide the mechanistic context for the differential recovery trajectories documented in Table 3.

Six-month trajectories diverged across therapy classes but should be interpreted in light of the baseline risk differences shown in Table 1. LVEF improved by 16.5 ± 4.9 percentage points in SGLT-2 inhibitor-treated patients and by 14.5 ± 5.1 points in the GLP-1 receptor agonist group, compared with 8.3 ± 6.1 points in the conventional-therapy group (*p* < 0.001 across groups). The absolute reduction in LV end-diastolic volume was larger in SGLT-2 inhibitor recipients than in conventional or DPP-4 inhibitor recipients. The protocol-defined reverse-remodeling rate was 59.1% in the SGLT-2 inhibitor group and 57.1% in the GLP-1 receptor agonist group, versus 37.5% in DPP-4 inhibitor and 30.4% in conventional-therapy patients. Myocardial work indices improved in all groups, with the largest mean gains in global constructive work and global work efficiency among SGLT-2 inhibitor and GLP-1 receptor agonist users. These findings demonstrate associations between contemporary cardiometabolic therapy exposure and recovery trajectories but do not establish class causality.

Over a median follow-up of 17 months (IQR 14–22), 45 patients (17.8%) experienced an MACE. Event rates differed across descriptive groups (*p* < 0.001 by log-rank test, Table 4 and Figure 4), but comparisons were limited by small event counts within the diabetic therapy strata and by substantial baseline imbalance. Conventional-therapy patients had the highest crude event burden (39.1%), driven principally by heart failure rehospitalization. The lower crude event rate among SGLT-2 inhibitor users should not be interpreted as complete offset of the prognostic effect of diabetes. SGLT-2 inhibitor patients had only two events, were more frequently discharged on guideline-directed medical therapy than the non-diabetic reference group, and had less residual unrevascularized multivessel disease than conventional-therapy patients. In parsimonious sensitivity models limited to Killip class and baseline GWE, the direction of the conventional-therapy signal was similar but confidence intervals widened substantially, reinforcing the exploratory nature of clinical outcome analyses.

Within the diabetic subgroup of 75 patients, univariable logistic regression was used as the primary modeling approach for reverse remodeling (Table 5). Higher baseline GWE was the most consistent predictor of reverse remodeling, and SGLT-2 inhibitor exposure was associated with higher odds of reverse remodeling relative to conventional therapy. The adjusted estimates are presented only as a parsimonious sensitivity analysis and should not be considered definitive because the number of patients per therapy stratum was small. GLP-1 receptor agonist exposure showed a directionally similar but imprecise association, whereas DPP-4 inhibitor estimates overlapped unity.

Sex-stratified analyses (Table 6) confirmed that the male-favorable remodeling pattern described in the parent cohort held within each therapy class, but with different magnitudes. Men consistently achieved larger absolute reductions in LVEDV than women across all five groups, with the largest absolute sex gap observed in the non-diabetic reference (−18.9 mL vs. −12.6 mL) and in the conventional-therapy group (−14.9 mL vs. −8.3 mL). The sex disparity in reverse-remodeling rates was pronounced in the conventional-therapy group (41.7% in men vs. 18.2% in women), whereas it narrowed substantially in SGLT-2i recipients (62.5% vs. 50.0%). A formal test of the therapy × sex interaction was not significant at the overall level (*p* = 0.341), indicating that the hierarchy of therapeutic benefit did not fundamentally reverse between sexes. However, within the non-diabetic reference, men showed a significantly larger ΔGWE than women (*p* = 0.041), whereas in SGLT-2i, GLP-1 RA, and DPP-4i recipients, the sex-related difference in ΔGWE was attenuated (interaction *p* ≥ 0.28 in each). Conventional-therapy patients showed a borderline interaction (*p* = 0.067). These findings tentatively suggest that the female disadvantage in energetic recovery after STEMI, documented previously in our parent cohort, is attenuated, though not abolished, by pre-admission treatment with the two most cardioprotective hypoglycemic drug classes.

**Figure 1:**
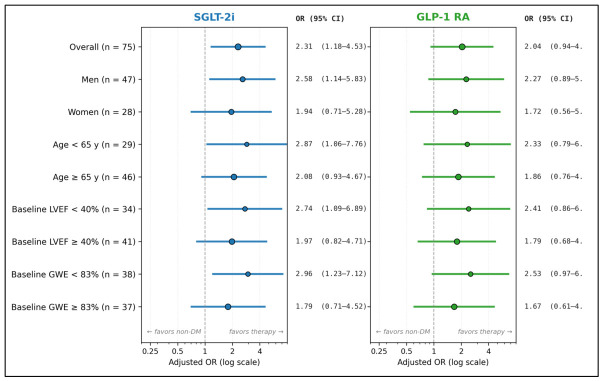
Exploratory odds ratios (95% CI) for reverse LV remodeling across pre-admission hypoglycemic therapy classes in the overall diabetic cohort and in sex- and age-based subgroups. Estimates are imprecise and should not be interpreted as confirmatory treatment effects.

**Figure 2:**
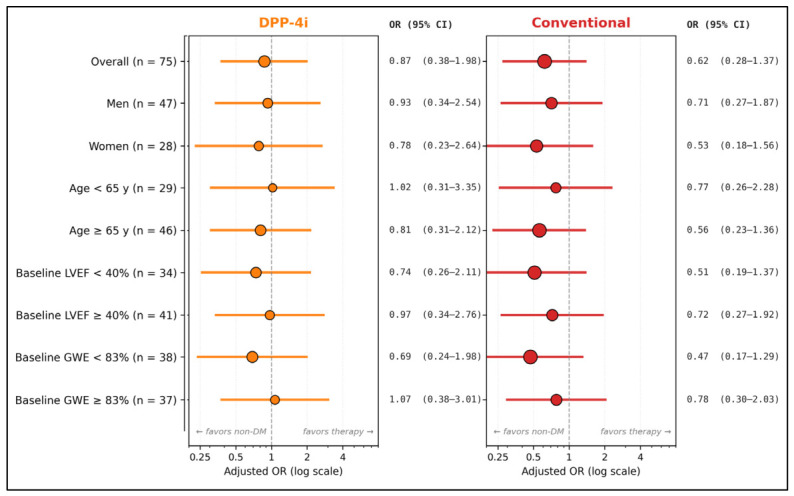
Exploratory odds ratios (95% CI) for reverse LV remodeling across pre-admission hypoglycemic therapy classes in baseline LVEF- and GWE-based subgroups. Estimates are descriptive only because of small subgroup sizes and multiplicity.

Subgroup forest plots are presented as exploratory analyses in Figure 1 and Figure 2. These estimates were retained only to show consistency of direction across clinically relevant strata and were not used for inference because several strata contained very small numbers of treated patients. Accordingly, multiplicity and imprecision are explicitly acknowledged, and subgroup odds ratios should be interpreted as hypothesis-generating.

**Figure 3:**
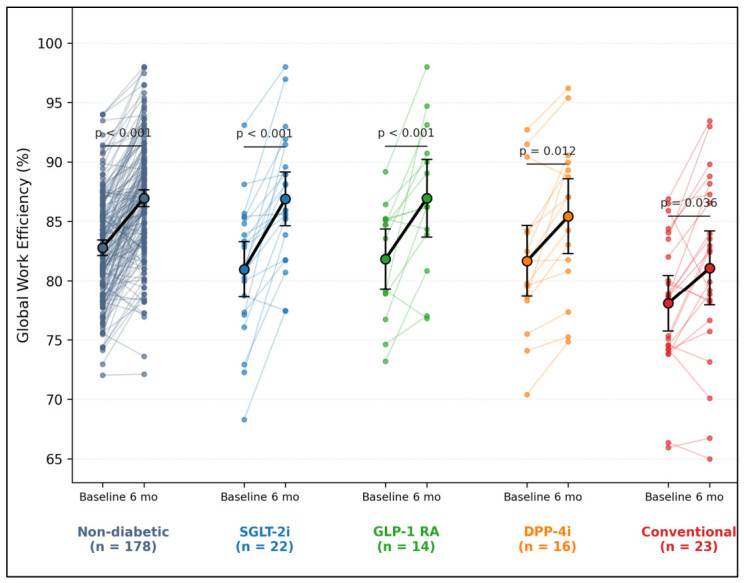
Paired within-patient trajectories of global work efficiency (GWE) from baseline to six months, stratified by pre-admission hypoglycemic therapy class. Thin translucent lines represent individual patients; thick black lines connect group means with 95% confidence intervals. Paired-sample *t*-test *p*-values are shown above each group.

Figure 3 shows that within-patient GWE gains were largest in the SGLT-2 inhibitor and GLP-1 receptor agonist groups, intermediate in the non-diabetic reference group, and attenuated in the DPP-4 inhibitor and conventional-therapy groups. Paired tests confirmed statistically significant within-group changes, but between-group interpretation remains limited by confounding by indication and post-discharge treatment differences.

Table 7 reports an exploratory propensity-score-matched comparison of the 22 SGLT-2 inhibitor users against 44 non-SGLT-2 inhibitor diabetic controls. Matching improved balance for most measured covariates, but residual imbalance remained for baseline LVEF and symptom-to-balloon time, and the effective sample size was small. Structural and myocardial work recovery estimates remained directionally favorable for SGLT-2 inhibitor exposure in the matched set, but these findings should be regarded as sensitivity analyses rather than confirmatory evidence. The MACE comparison was particularly underpowered and was not used to support clinical conclusions (Table 8).

**Figure 4:**
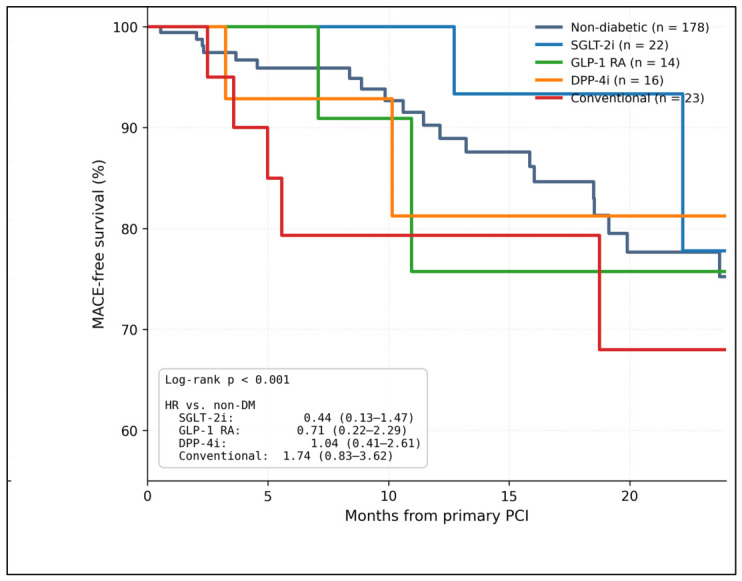
Kaplan–Meier curves for MACE-free survival by therapy group, with log-rank *p*-value and unadjusted Cox hazard ratios against the non-diabetic descriptive reference group.

Figure 4 presents unadjusted MACE-free survival curves. The curves separate early, but because the number of events in the SGLT-2 inhibitor, GLP-1 receptor agonist, and DPP-4 inhibitor groups was very small, the survival analysis is presented as descriptive and exploratory rather than as evidence of therapy-mediated clinical benefit.

## 4. Discussion

In this analysis of 253 reperfused STEMI patients with baseline LVEF ≤ 50%, pre-admission hypoglycemic therapy class was associated with six-month structural and energetic recovery. SGLT-2 inhibitor-treated patients had the highest reverse-remodeling rate, the largest reductions in LV volumes, and the greatest improvement in global work efficiency, while GLP-1 receptor agonist users showed a directionally similar but less precise recovery profile. The DPP-4 inhibitor and conventional-therapy groups showed smaller gains. Because baseline risk differed substantially across therapy classes, especially for diabetes duration, HbA1c, renal function, Killip class, NT-proBNP, multivessel coronary disease, and discharge medical therapy, these findings should be interpreted as hypothesis-generating associations rather than evidence of a class-related treatment effect. The present results complement the parent STEMI cohort in which baseline myocardial work efficiency emerged as a key predictor of later reverse remodeling after successful primary PCI [17,18,19,20,21,22].

The biologic plausibility of a favorable SGLT-2 inhibitor signal remains substantial, but the current AMI-specific literature supports cautious interpretation. Mechanistic work suggests that SGLT-2 inhibitors may reduce preload and afterload through natriuresis, improve myocardial energetics through substrate shifts, modulate sodium–hydrogen exchange, and attenuate inflammation and interstitial fibrosis [12,23,24,25]. However, the updated 2025 meta-analysis of early SGLT-2 inhibitor initiation after acute MI found reduced hospitalization for heart failure and MACEs in frequentist analyses, while Bayesian estimates remained uncertain and mortality endpoints were not significantly reduced [26,27,28,29,30,31]. The recent randomized dapagliflozin STEMI fibrosis trial further emphasizes that structural and tissue-level endpoints after primary PCI require prospective validation [32]. Therefore, the larger improvements in Delta LVEDV, Delta LVESV, and Delta GWE observed in our SGLT-2 inhibitor group are clinically coherent but cannot be separated from treatment-selection patterns in this observational dataset.

The GLP-1 receptor agonist findings deserve separate consideration. Although the sample size was smaller and confidence intervals were wider, GLP-1 receptor agonist users demonstrated a recovery trajectory approaching that of SGLT-2 inhibitor users, with high reverse-remodeling rates and improvements in both LVEF and GWE. This pattern is consistent with earlier reports of improved ventricular function after liraglutide exposure and with cardiovascular-outcome evidence supporting GLP-1 receptor agonist use in patients with T2DM at elevated atherosclerotic risk [13,26]. By contrast, the weaker DPP-4 inhibitor signal is concordant with the more neutral echocardiographic literature for DPP-4 inhibition [14]. These comparisons remain associative and should not be interpreted as a definitive hierarchy of post-STEMI therapy effects.

Myocardial work analysis adds a mechanistic layer to the interpretation of these findings. Pressure–strain loop-derived indices integrate myocardial deformation with afterload and therefore capture clinically relevant performance that conventional LVEF alone may underestimate [16,27]. Prior STEMI studies have shown that lower GWE and lower myocardial work index values identify patients at increased risk of adverse remodeling and poorer long-term outcomes [20,28]. In the present study, the exploratory mediation analysis suggested that approximately 41.6% of the association between SGLT-2 inhibitor exposure and LVEDV reduction was statistically linked to improvement in GWE. This finding is hypothesis-generating only because treatment allocation was observational and Delta GWE and Delta LVEDV were measured over the same period. It should be viewed as support for future mechanistic studies rather than as evidence of causal mediation.

The clinical outcome findings should also be interpreted cautiously. Conventional-therapy patients had the highest crude MACE burden, especially heart failure rehospitalization, but they also had a higher-risk presentation, more residual unrevascularized multivessel disease, and less favorable glucometabolic control. Recent evidence indicates that glucometabolic markers, particularly the stress hyperglycemia ratio, improve risk stratification in ACS patients undergoing PCI and are associated with periprocedural myocardial infarction and adverse long-term cardiovascular outcomes [32,33]. The gradient in HbA1c, fasting glucose, and acute presentation severity in our cohort therefore reinforces the need to account for metabolic risk rather than attributing event differences to drug class alone. The present data do not justify therapy-class selection on the basis of echocardiography alone, but they support prospective studies in which myocardial work indices are incorporated as enrichment biomarkers and mechanistic endpoints.

### Study Limitations

Several limitations temper our conclusions. First, this is a single-center observational cohort, and therapy assignment was not randomized; confounding by indication, prescriber preferences, socioeconomic factors, diabetes severity, and access to contemporary cardiometabolic therapy cannot be fully excluded. Second, the diabetic subgroup totaled only 75 patients, with 22 SGLT-2 inhibitor users and 14 GLP-1 receptor agonist users; this limited the stability of regression, survival, propensity-score, mediation, and subgroup analyses. Accordingly, high-dimensional Cox interaction modeling was removed, and adjusted models were simplified and reframed as exploratory sensitivity analyses. Third, clinically important baseline imbalances were present, including diabetes duration, HbA1c, renal function, Killip class, NT-proBNP, multivessel coronary disease, residual revascularization status, and discharge guideline-directed medical therapy. Fourth, the non-diabetic group was biologically distinct and should be viewed only as a descriptive reference, not as a therapy comparator. Fifth, myocardial work indices were estimated non-invasively using brachial cuff pressure as a surrogate for peak LV pressure; systematic measurement error cannot be fully excluded. Sixth, post-discharge therapy modifications occurred during follow-up; the primary analysis used pre-admission therapy class by design, but this limits interpretation of six-month remodeling. Finally, the median follow-up of 17 months and the small number of clinical events preclude firm conclusions about long-term MACE or mortality differences.

## 5. Conclusions

In this cohort of reperfused STEMI patients with reduced baseline LVEF, pre-admission glucose-lowering therapy class was associated with distinct patterns of LV reverse remodeling and myocardial work recovery. Exposure to SGLT-2 inhibitors and GLP-1 receptor agonists was associated with more favorable six-month structural and energetic recovery than DPP-4 inhibitors or conventional therapy, but these associations were observed in a small, non-randomized diabetic subgroup with substantial baseline imbalance. The clinical outcomes and mediation findings are exploratory and hypothesis-generating. Larger prospective studies are needed to determine whether myocardial work indices can guide post-STEMI risk stratification or serve as mechanistic endpoints for cardiometabolic therapy trials.

## Figures and Tables

**Table 1:** Baseline demographic, clinical, laboratory, revascularization, and discharge treatment characteristics across pre-admission hypoglycemic therapy groups.

Variable	Non-Diabetic (*n* = 178)	SGLT-2i (*n* = 22)	GLP-1 RA (*n* = 14)	DPP-4i (*n* = 16)	Conventional (*n* = 23)	*p*-Value
Age (years), mean ± SD	65.8 ± 13.4	68.2 ± 10.7	66.9 ± 11.3	71.3 ± 9.8	69.6 ± 10.4	0.183
Women, *n* (%)	63 (35.4)	6 (27.3)	5 (35.7)	6 (37.5)	11 (47.8)	0.476
BMI (kg/m^2^), mean ± SD	27.4 ± 4.3	30.1 ± 4.7	31.3 ± 5.2	28.6 ± 4.1	28.9 ± 4.6	0.004
Hypertension, *n* (%)	131 (73.6)	19 (86.4)	12 (85.7)	14 (87.5)	19 (82.6)	0.296
Hypercholesterolemia, *n* (%)	124 (69.7)	18 (81.8)	11 (78.6)	13 (81.3)	17 (73.9)	0.524
Current smoker, *n* (%)	87 (48.9)	8 (36.4)	5 (35.7)	6 (37.5)	9 (39.1)	0.412
Prior stroke, *n* (%)	11 (6.2)	2 (9.1)	1 (7.1)	2 (12.5)	3 (13.0)	0.613
DM duration (years), median [IQR]	—	6.3 [3.1–9.4]	7.1 [4.2–11.7]	8.4 [5.3–13.2]	9.8 [6.1–14.6]	0.038
HbA1c (%), mean ± SD	5.6 ± 0.4	7.3 ± 1.1	7.5 ± 1.3	7.7 ± 1.2	8.2 ± 1.6	<0.001
Fasting glucose (mg/dL)	102.4 ± 14.3	143.7 ± 38.2	149.6 ± 41.3	156.3 ± 44.1	173.8 ± 52.7	<0.001
eGFR (mL/min/1.73 m^2^)	52.3 ± 23.7	51.8 ± 19.4	49.6 ± 21.3	44.2 ± 18.7	46.1 ± 22.4	0.041
Killip class ≥ III, *n* (%)	52 (29.2)	6 (27.3)	4 (28.6)	7 (43.8)	13 (56.5)	0.023
NT-proBNP (ng/L), median [IQR]	210 [80–440]	240 [95–510]	235 [110–480]	330 [125–680]	410 [180–780]	0.014
Peak hs-TnI (ng/L)	14,360 ± 8740	15,820 ± 9210	14,960 ± 8830	17,420 ± 10,130	18,740 ± 11,260	0.189
Symptom-to-balloon (min)	194 [138–241]	206 [149–253]	203 [146–248]	221 [162–278]	238 [174–287]	0.067
LAD culprit vessel, *n* (%)	98 (55.1)	13 (59.1)	8 (57.1)	10 (62.5)	13 (56.5)	0.931
Multivessel CAD, *n* (%)	71 (39.9)	12 (54.5)	9 (64.3)	11 (68.8)	15 (65.2)	0.009
ACEI/ARB at discharge, *n* (%)	118 (66.3)	19 (86.4)	12 (85.7)	14 (87.5)	19 (82.6)	0.031
Beta-blocker at discharge, *n* (%)	137 (77.0)	20 (90.9)	13 (92.9)	14 (87.5)	20 (87.0)	0.184
MRA at discharge, *n* (%)	58 (32.6)	12 (54.5)	8 (57.1)	7 (43.8)	13 (56.5)	0.038
High-intensity statin, *n* (%)	147 (82.6)	20 (90.9)	13 (92.9)	14 (87.5)	20 (87.0)	0.681

*p*-values from one-way ANOVA (continuous normally distributed), Kruskal–Wallis test (non-normal), or chi-square/Fisher exact test (categorical). Significant omnibus tests were followed by Tukey HSD, Dunn–Holm, or false-discovery-rate-corrected pairwise comparisons, as appropriate. The main post hoc patterns were as follows: conventional therapy had higher HbA1c and fasting glucose than SGLT-2 inhibitor therapy; conventional therapy had higher Killip class and NT-proBNP than SGLT-2 inhibitor and GLP-1 receptor agonist therapies; and multivessel coronary artery disease was more common in diabetic therapy strata than in the non-diabetic reference group. SGLT-2i = sodium–glucose co-transporter-2 inhibitor; GLP-1 RA = glucagon-like peptide-1 receptor agonist; DPP-4i = dipeptidyl peptidase-4 inhibitor; BMI = body mass index; HbA1c = glycated hemoglobin; eGFR = estimated glomerular filtration rate; NT-proBNP = N-terminal pro B-type natriuretic peptide; hs-TnI = high-sensitivity troponin I; LAD = left anterior descending artery; CAD = coronary artery disease; ACEI/ARB = angiotensin-converting enzyme inhibitor/angiotensin receptor blocker; MRA = mineralocorticoid receptor antagonist.

**Table 2:** Baseline echocardiographic parameters and myocardial work indices across therapy groups.

Parameter	Non-Diabetic (*n* = 178)	SGLT-2i (*n* = 22)	GLP-1 RA (*n* = 14)	DPP-4i (*n* = 16)	Conventional (*n* = 23)	*p*-Value
LVEF (%)	41.8 ± 6.7	40.3 ± 7.2	41.1 ± 6.9	41.6 ± 6.4	40.9 ± 7.1	0.871
LVEDV (mL)	126.3 ± 27.8	131.7 ± 29.6	128.4 ± 26.9	129.2 ± 28.3	133.8 ± 31.4	0.563
LVESV (mL)	73.8 ± 18.2	78.7 ± 19.7	75.6 ± 18.1	75.5 ± 18.9	79.2 ± 21.3	0.647
Stroke volume index (mL/m^2^)	41.3 ± 10.6	39.8 ± 11.2	40.1 ± 10.4	39.6 ± 11.3	38.4 ± 12.1	0.711
E/e′ ratio	10.2 ± 3.1	11.7 ± 3.4	11.3 ± 3.2	12.4 ± 3.8	13.1 ± 4.2	0.002
WMSI	2.21 ± 0.23	2.26 ± 0.24	2.23 ± 0.22	2.29 ± 0.25	2.31 ± 0.27	0.374
GLS (%)	−17.8 ± 3.4	−17.2 ± 3.6	−17.6 ± 3.3	−16.9 ± 3.8	−16.3 ± 3.9	0.247
GWI (mmHg%)	1712 ± 312	1638 ± 347	1681 ± 329	1594 ± 361	1521 ± 374	0.071
GCW (mmHg%)	1867 ± 172	1792 ± 184	1823 ± 176	1761 ± 193	1714 ± 206	0.017
GWW (mmHg%)	208.3 ± 41.6	224.7 ± 47.3	218.6 ± 43.8	236.4 ± 49.2	247.8 ± 52.6	0.003
GWE (%)	83.1 ± 5.2	81.7 ± 5.6	82.3 ± 5.4	80.6 ± 5.9	80.1 ± 6.1	0.048

Values are mean ± SD. *p*-values from one-way ANOVA. LVEF = left ventricular ejection fraction; LVEDV/LVESV = LV end-diastolic/end-systolic volume; E/e′ = ratio of early mitral inflow to annular tissue Doppler velocity; WMSI = wall motion score index; GLS = global longitudinal strain; GWI = global work index; GCW = global constructive work; GWW = global wasted work; GWE = global work efficiency.

**Table 3:** Six-month echocardiographic changes and reverse remodeling rates by therapy group.

Parameter/Outcome	Non-Diabetic (*n* = 178)	SGLT-2i (*n* = 22)	GLP-1 RA (*n* = 14)	DPP-4i (*n* = 16)	Conventional (*n* = 23)	*p*-Value
ΔLVEF (percentage points)	+11.6 ± 5.3	+16.5 ± 4.9	+14.5 ± 5.1	+9.7 ± 5.4	+8.3 ± 6.1	<0.001
ΔLVEDV (mL)	−16.7 ± 8.4	−27.4 ± 9.1	−21.6 ± 8.7	−11.9 ± 7.8	−12.2 ± 9.3	<0.001
ΔLVESV (mL)	−21.9 ± 7.2	−33.8 ± 8.3	−28.1 ± 7.6	−18.5 ± 6.9	−17.2 ± 8.4	<0.001
ΔGLS (percentage points)	−2.1 ± 1.7	−3.6 ± 1.9	−2.9 ± 1.8	−1.4 ± 1.6	−1.1 ± 2.1	<0.001
ΔGWI (mmHg%)	+193 ± 118	+287 ± 131	+241 ± 124	+132 ± 109	+98 ± 127	<0.001
ΔGCW (mmHg%)	+271 ± 97	+394 ± 108	+342 ± 102	+213 ± 94	+174 ± 116	<0.001
ΔGWW (mmHg%)	−18.4 ± 24.7	−32.6 ± 27.1	−24.1 ± 25.3	−9.8 ± 23.9	−4.3 ± 28.4	<0.001
ΔGWE (percentage points)	+4.1 ± 3.6	+6.8 ± 3.4	+5.9 ± 3.5	+2.7 ± 3.8	+1.9 ± 4.0	<0.001
Reverse remodeling, *n* (%)	76 (42.7)	13 (59.1)	8 (57.1)	6 (37.5)	7 (30.4)	0.019
LVEF recovery ≥ 10 pp, *n* (%)	94 (52.8)	17 (77.3)	10 (71.4)	7 (43.8)	8 (34.8)	0.005

Delta values represent six-month follow-up minus baseline. *p*-values from one-way ANOVA for continuous variables and chi-square tests for proportions. Significant omnibus tests were followed by Tukey or Dunn–Holm pairwise comparisons; the principal post hoc contrasts showed larger Delta LVEF, Delta LVEDV, Delta LVESV, Delta GCW, and Delta GWE improvements in SGLT-2 inhibitor and GLP-1 receptor agonist groups than in conventional therapy, whereas DPP-4 inhibitor estimates were intermediate and generally not significantly different from conventional therapy after correction. Reverse remodeling was defined as ≥ 15% reduction in LV end-diastolic volume.

**Table 4:** Clinical outcomes over a median follow-up of 17 months and unadjusted survival comparisons.

Therapy Group	MACEs, *n* (%)	All-Cause Death, *n* (%)	HF Rehospitalization, *n* (%)	Reinfarction, *n* (%)	Unadjusted HR (95% CI) †
Non-diabetic (*n* = 178)	29 (16.3)	9 (5.1)	14 (7.9)	6 (3.4)	1.00 (reference)
SGLT-2i (*n* = 22)	2 (9.1)	0 (0.0)	1 (4.5)	1 (4.5)	0.44 (0.13–1.47)
GLP-1 RA (*n* = 14)	2 (14.3)	0 (0.0)	1 (7.1)	1 (7.1)	0.71 (0.22–2.29)
DPP-4i (*n* = 16)	3 (18.8)	1 (6.3)	2 (12.5)	0 (0.0)	1.04 (0.41–2.61)
Conventional (*n* = 23)	9 (39.1)	3 (13.0)	5 (21.7)	1 (4.3)	1.74 (0.83–3.62)
Overall *p*-value (log-rank)	*p* < 0.001	—	—	—	—

† Hazard ratios are unadjusted Cox estimates and are shown to match the Kaplan–Meier estimates in Figure 4. Because the number of MACEs within individual diabetic therapy strata was small, adjusted Cox models were retained only as exploratory sensitivity analyses and are not used as primary evidence. Non-diabetic patients are shown as a descriptive reference group, not as a therapy comparator. MACE = major adverse cardiac event (composite of all-cause death, heart failure [HF] rehospitalization, and non-fatal reinfarction).

**Table 5:** Univariable logistic regression and parsimonious adjusted sensitivity analysis for reverse LV remodeling in the diabetic subgroup (*n* = 75).

Predictor	Univariable OR (95% CI)	*p*-Value	Multivariable OR (95% CI)	*p*-Value
Age (per 10-year increase)	0.76 (0.54–1.07)	0.114	0.82 (0.56–1.21)	0.323
Female sex	0.52 (0.21–1.29)	0.157	—	—
HbA1c (per 1% increase)	0.68 (0.52–0.89)	0.005	0.79 (0.58–1.07)	0.127
Baseline LVEF (per 5-point increase)	1.37 (1.02–1.84)	0.037	1.26 (0.88–1.80)	0.204
Baseline GWE (per 5-point increase)	1.96 (1.37–2.81)	<0.001	1.78 (1.21–2.62)	0.003
Baseline GCW (per 100 mmHg% increase)	1.41 (1.14–1.75)	0.002	1.23 (0.94–1.61)	0.132
Killip class ≥ III	0.42 (0.18–0.97)	0.042	0.58 (0.21–1.59)	0.289
NT-proBNP (per log_10_ increase)	0.57 (0.36–0.89)	0.014	0.71 (0.42–1.19)	0.194
Symptom-to-balloon > 180 min	0.48 (0.22–1.05)	0.066	0.61 (0.26–1.44)	0.258
Multivessel CAD	0.61 (0.26–1.42)	0.250	—	—
SGLT-2i therapy (vs. conventional)	3.31 (1.04–10.52)	0.043	4.17 (1.18–14.72)	0.027
GLP-1 RA therapy (vs. conventional)	3.05 (0.87–10.64)	0.081	3.62 (0.94–13.93)	0.061
DPP-4i therapy (vs. conventional)	1.38 (0.36–5.24)	0.639	1.53 (0.38–6.21)	0.554

Outcome: reverse remodeling (≥15% LVEDV reduction at 6 months). Univariable estimates are the main analysis. The adjusted column is an exploratory parsimonious sensitivity model limited to therapy class, baseline GWE, and Killip class because of the small diabetic subgroup size. Estimates should be interpreted as association measures rather than causal treatment effects. OR = odds ratio; CI = confidence interval.

**Table 6:** Sex-stratified reverse remodeling rates, myocardial work changes, and MACE rates within each therapy group.

Therapy Group	ΔLVEDV (mL), Men	ΔLVEDV (mL), Women	RR Rate, Men (%)	RR Rate, Women (%)	Interaction ΔGWE by Sex (*p*)
Non-diabetic	−18.9 ± 8.1	−12.6 ± 8.7	48.7	32.4	0.041
SGLT-2i	−29.8 ± 8.7	−21.3 ± 9.4	62.5	50.0	0.376
GLP-1 RA	−23.7 ± 8.2	−18.1 ± 9.1	66.7	40.0	0.412
DPP-4i	−13.4 ± 7.6	−10.1 ± 8.1	40.0	33.3	0.283
Conventional	−14.9 ± 9.1	−8.3 ± 9.2	41.7	18.2	0.067
Interaction therapy × sex (overall)	—	—	—	—	0.341

Values are mean ± SD or percentages. Interaction *p*-values derive from mixed-effects models with a therapy × sex product term and subject-level random intercepts; the overall interaction was tested in a single model including all therapy groups. RR = reverse remodeling.

**Table 7:** Exploratory propensity-score-matched comparison of SGLT-2 inhibitor users versus non-SGLT-2 inhibitor diabetic patients (1:2 nearest-neighbor matching, caliper 0.2 SD of the logit).

Covariate/Outcome	SGLT-2i (*n* = 22)	Matched Controls (*n* = 44)	SMD Before	SMD After	Between-Group Effect (95% CI)/*p*
Age (years)	68.2 ± 10.7	69.4 ± 10.3	0.142	0.061	—
Female sex, *n* (%)	6 (27.3)	13 (29.5)	0.097	0.051	—
HbA1c (%)	7.3 ± 1.1	7.4 ± 1.2	0.274	0.083	—
eGFR (mL/min/1.73 m^2^)	51.8 ± 19.4	50.3 ± 20.1	0.198	0.076	—
Killip class ≥ III, *n* (%)	6 (27.3)	13 (29.5)	0.231	0.049	—
log_10_ NT-proBNP	2.41 ± 0.38	2.44 ± 0.41	0.263	0.074	—
Baseline LVEF (%)	40.3 ± 7.2	41.1 ± 6.8	0.114	0.114	—
Baseline GWE (%)	81.7 ± 5.6	81.2 ± 5.8	0.089	0.088	—
Symptom-to-balloon (min)	206 ± 71	217 ± 68	0.181	0.157	—
Multivessel CAD, *n* (%)	12 (54.5)	24 (54.5)	0.204	0.000	—
ΔLVEDV (mL)	−27.4 ± 9.1	−12.3 ± 8.9	—	—	−15.1 mL (−20.1 to −10.1), *p* < 0.001
ΔLVEF (percentage points)	+16.5 ± 4.9	+9.1 ± 5.7	—	—	+7.4 pp (4.6 to 10.2), *p* < 0.001
ΔGWE (percentage points)	+6.8 ± 3.4	+2.3 ± 3.9	—	—	+4.5 pp (2.6 to 6.4), *p* < 0.001
Reverse remodeling, *n* (%)	13 (59.1)	11 (25.0)	—	—	RR 2.36 (1.24–4.50), *p* = 0.006
MACE at 17 months, *n* (%)	2 (9.1)	11 (25.0)	—	—	HR 0.36 (0.08–1.63), *p* = 0.183

Propensity score derived from logistic regression on baseline covariates selected a priori. SMD = standardized mean difference; values < 0.10 indicate adequate balance. Residual imbalance persisted for baseline LVEF and symptom-to-balloon time; therefore, this analysis is reported only as exploratory sensitivity evidence. No definitive clinical outcome inference was drawn from the matched MACE comparison because of the limited number of events.

**Table 8:** Exploratory mediation analysis of the association between SGLT-2 inhibitor exposure, six-month change in global work efficiency, and change in LVEDV in the diabetic subgroup (*n* = 75).

Effect Component	Point Estimate (mL)	95% Bootstrap CI	*p*-Value	Proportion of Total Effect (%)
Estimated indirect effect via ΔGWE	−6.24	−9.38 to −3.41	<0.001	41.6
Estimated direct effect of SGLT-2i	−8.76	−14.03 to −3.52	<0.001	58.4
Total effect	−15.00	−20.89 to −9.37	<0.001	100
Proportion explained (indirect/total)	0.416	0.211 to 0.682	<0.001	—
Sensitivity: residual correlation at which indirect effect = 0	−0.38	—	—	—
Sensitivity: R^2^ of unmeasured confounder at indirect effect = 0	0.19	—	—	—
Mediation in men (*n* = 47)	−7.41	−10.92 to −4.16	<0.001	44.3
Mediation in women (*n* = 28)	−4.37	−8.12 to −1.24	0.008	36.8
Mediation in baseline GWE < 83% (*n* = 38)	−8.67	−13.01 to −4.73	<0.001	49.2
Mediation in baseline GWE ≥ 83% (*n* = 37)	−2.91	−6.04 to −0.16	0.038	27.1

## Data Availability

The anonymized data supporting the findings of this study are available from the corresponding authors on reasonable request, subject to institutional data-sharing agreements.
